# The Production of Extracellular Proteins Is Regulated by Ribonuclease III via Two Different Pathways in *Staphylococcus aureus*

**DOI:** 10.1371/journal.pone.0020554

**Published:** 2011-05-31

**Authors:** Yu Liu, Jie Dong, Na Wu, Yaping Gao, Xin Zhang, Chunhua Mu, Ningsheng Shao, Ming Fan, Guang Yang

**Affiliations:** Beijing Institute of Basic Medical Sciences, Beijing, People's Republic of China; University of Edinburgh, United Kingdom

## Abstract

*Staphylococcus aureus* ribonuclease III belongs to the enzyme family known to degrade double-stranded RNAs. It has previously been reported that RNase III cannot influence cell growth but regulates virulence gene expression in *S. aureus*. Here we constructed an RNase III inactivation mutant (*Δrnc*) from *S. aureus* 8325-4. It was found that the extracellular proteins of *Δrnc* were decreased. Furthermore, we explored how RNase III regulated the production of the extracellular proteins in *S. aureus.* We found during the lag phase of the bacterial growth cycle RNase III could influence the extracellular protein secretion via regulating the expression of *secY2*, one component of accessory secretory (sec) pathway. After *S. aureus* cells grew to exponential phase, RNase III can regulate the expression of extracellular proteins by affecting the level of RNAIII. Further investigation showed that the mRNA stability of *secY2* and RNAIII was affected by RNase III. Our results suggest that RNase III could regulate the pathogenicity of *S. aureus* by influencing the level of extracellular proteins via two different ways respectively at different growth phases.

## Introduction

Ribonuclease III (RNase III) is a double-stranded endoribonuclease, which has been classified into three main groups on the basis of their domain organization [1]. Bacterial RNase III belongs to Group I family, which contains only one characteristic ribonuclease domain and one dsRNA-binding domain (dsRBD) [1], [2].

RNase III has been thought to be important in *Escherichia coli* (*E. coli*) because it is involved in the process of both 16s and 23s rRNAs from a 30s precursor [3]. Further, it is found that RNase III has an additional function to degrade mRNA with the mediation of trans-acting antisense RNA in *E. coli* [4], [5]. Although *Staphylococcus aureus* (*S. aureus*) RNase III seems to play a minor role in the formation of 30S rRNA [6], it is reported that RNase III can induce mRNA degradation mediated by RNAIII, which is an important regulator of the quorum sensing system (agr) [7], [8]. It is reported that RNAIII generally acts by an antisense base pairing mechanism [7], [8], and regulates many target genes via its control of a repressor protein gene called *rot*, a member of the sarA family of transcriptional regulators [8], [9], [10]. RNase III can degrade the target mRNAs of RNAIII but not hydrolyze RNAIII [7], [8]. It suggests that RNase III should be essential for virulence gene regulation in *S. aureus*. But the biological function of RNase III of *S. aureus* is still unclear.

The secreted proteins play an important role for the pathogenicity of *S. aureus* [11]. The majority of exported proteins are transported from the cytoplasm via the general secretory (sec) pathway(including *secA/Y/E/G*) in gram positive bacteria [12]. In addition, *S. aureus* contains an accessory Sec pathway involving the SecA2 and SecY2 proteins [12], [13], [14]. In contrast to the general sec pathway, SecY2 and SecA2 are not involved in the viability of *S. aureus* [12], [14]. In some pathogenic gram-positive bacteria, SecY2 is required for the transport of certain proteins related to virulence[12], [15], [16], [17]. However, SecY2-related secretomes have yet to be studied extensively [14].

We hereby tried to investigate the biological function of RNase III in *S. aureus* by constructing an RNase III inactivation mutant (*Δrnc*). Compared with its parent strain, both the extracellular proteins and the pathogenicity of *Δrnc* were reduced. In this report, we show that RNase III could influence the production of extracellular proteins of *S. aureus* by separately regulating the expression level of *secY2* and *RNAIII* via respective mechanisms at the different phases.

## Results

### Inactivation of RNase III did not influence the growth of *S. aureus*

**Figure 1:**
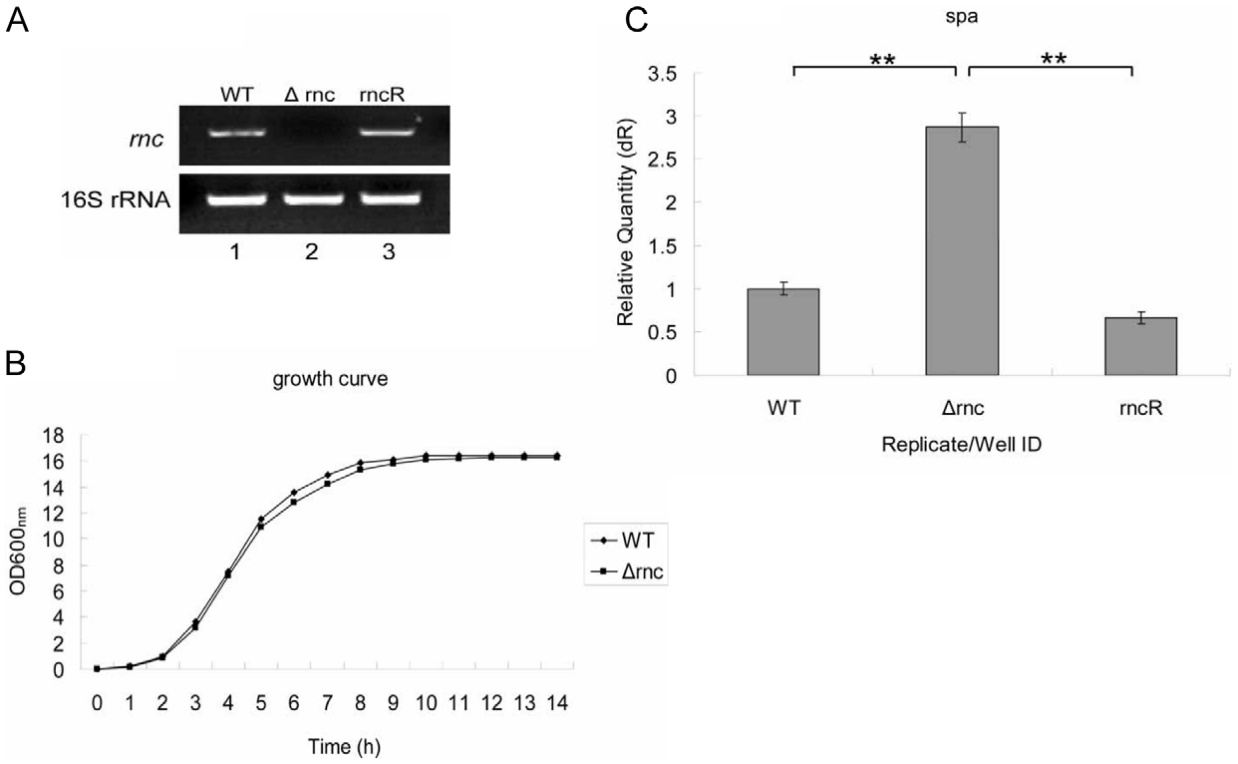
Detection of RNase III inactive mutant. **A: Verification of RNase III inactive mutant by RT-PCR.** Total RNA of cells was extract and used as the template to amplify the *rnc* gene. In *Δrnc* strain, the *rnc* mRNA could not be detected like WT and rncR because the kana gene was inserted into the *rnc* gene of *Δrnc* genome. 16s rRNA was used as the internal control. WT (wild type, *S. aureus* 8325-4), *Δrnc*(an RNase III inactivation mutant from 8325-4) and rncR (the restoration of RNase III activity in *Δrnc*). **B: The growth curves of** ***S. aureus*** **strains.** There is no significant difference between WT and *Δrnc*. WT: wild type, *S. aureus* 8325-4; *Δrnc*: an RNase III inactivation mutant from 8325-4. The experiment has been repeated for three times. **C: qRT-PCR quantification of the expression level of** ***spa***. The total RNA of the cells cultured for 6 h was extracted and the mRNA level of *spa* was detected by qRT-PCR. In the *Δrnc* strain, the level of *spa* mRNA was significantly increased compared with WT. WT: wild type, *S. aureus* 8325-4; *Δrnc*: an RNase III inactivation mutant from 8325-4; rncR: the restoration of RNase III activity in *Δrnc*. (**: P<0.01).

We constructed an RNase III inactivation strain (*Δrnc*) in 8325-4 with allelic homologous recombination. Then the mutant was verified by RT-PCR (figure 1A) using the primers (rncV1 and rncV2) as listed in the Table 1. To further observe the phenotype of *Δrnc*, the growth curves of *Δrnc* and its parent strain were measured. The result showed that there were no obvious differences observed between the wild type and mutation strains (figure 1B). In previous reports, RNase III could degrade the target mRNAs (*spa*) of RNAIII [7], so we tested the mRNA level of *spa* by real-time quantitative PCR. Compared with its parent strain, the level of *spa* significantly increased in *Δrnc* (figure 1C).

**Table 1:** Sequences of forward and reverse primers used in this study.

Primer/sequence	Oligonucleotide sequence (5′ to 3′)
Up-*rnc* F-EcoRI	CATCCGGAATTCATGTCTAAACAAAAGAA
Up-*rnc* R-KpnI	AACAAAATGA GGTACCAGGCGTGGTAGATT
Down- *rnc* F-KpnI	AATCTACCAC GCCTGGTACC TCATTTTGTT
Down- *rnc* R-SalI	ACACGCGTCGACTAGGCACTTTCAGCAGC
rs-rncF	GACTACGTGAATTCGACCGTTTAGGTGTA
rs-rncR	CATGCGTACTGCAGCTATTTAATTTGTTT
rncV1	CATCCGGAATTC TCGAGTTTTA TTAAT
rncV2	TTATAGGCAC TTTCAGCAGC
16s RT primerF	GCCTAATACATGCAAGT
16s RT primerR	CATGTTATCCGGCATTAG
spa RT primerF	AAGAAGATGGTAACGGAGTA
spa RT primerR	GTTGTACCGATGAATGGA
RNAIII RTprimerF	CCTAGATCAC AGAGATGTGA TGG
RNAIII RTprimerR	AATACATAGC ACTGAGTCCA AGG
efb RT primerF	AACATTAGCG GCAATAGG
efb RT primerR	TTTCGCTGCT GGTTTATT
Uefb-lacZF	GATATGCATGAATTCGACAATTTCCAATCT GTCTT
Uefb-lacZR	ATCATCGCGGATCCCCTTTCTTTTCTCTTG GCATGTTAAT TATCCTCCAA ATTATT
UefbSP-lacZR	CCAAGAGAAAAGAAAGGGGATCCGCGATGAT

### The extracellular proteins in the supernatant of *Δrnc* decreased significantly

**Figure 2:**
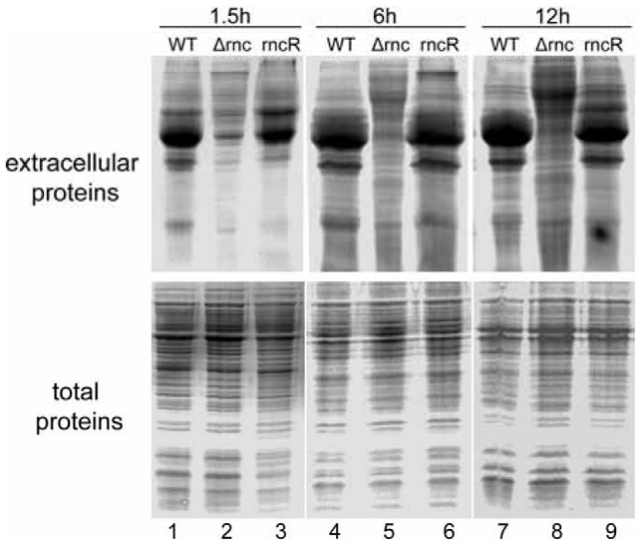
Detection of the protein profile from different phases of WT and *Δrnc*. Equal number of *S. aureus* cells was harvested at the indicated time points. The total proteins of whole-cell and supernatant proteins were extracted. The results showed that the supernatant proteins of the *Δrnc* were decreased significantly compared with WT, while the total proteins of whole-cell did not show the same change as the supernatant proteins. The experiment has been repeated for three times. 1,4,7: WT, wild type, *S. aureus* 8325-4; 2,5,8: *Δrnc*; 3,6,9: rncR.

The extracellular proteins play an important role for the pathogenicity of *S. aureus* [11]. We compared the profiles of the extracellular proteins from the same number of cells between *Δrnc* and its parent strain at the different growth phases. According to the growth curve, *S. aureus* cultured for 1.5 h, 6 h and 12 h is at the lag phase, exponential phase and stationary phase respectively. The proteins in the supernatant at different time points (1.5 h, 6 h, and 12 h) were extracted as described in Material and methods and the profile of the extracellular proteins in the supernatant was determined by SDS-PAGE. It was surprising that the extracellular proteins of *Δrnc* decreased significantly compared with its parent strain at three time points (figure 2). At the same time, we compared the total proteins of whole-cell between *Δrnc* and its parent strain. However, we did not find obvious changes in the total proteins (figure 2).

### A lower level of RNAIII in *Δrnc* led to reduction of extracellular proteins at 6 h and 12 h

**Figure 3:**
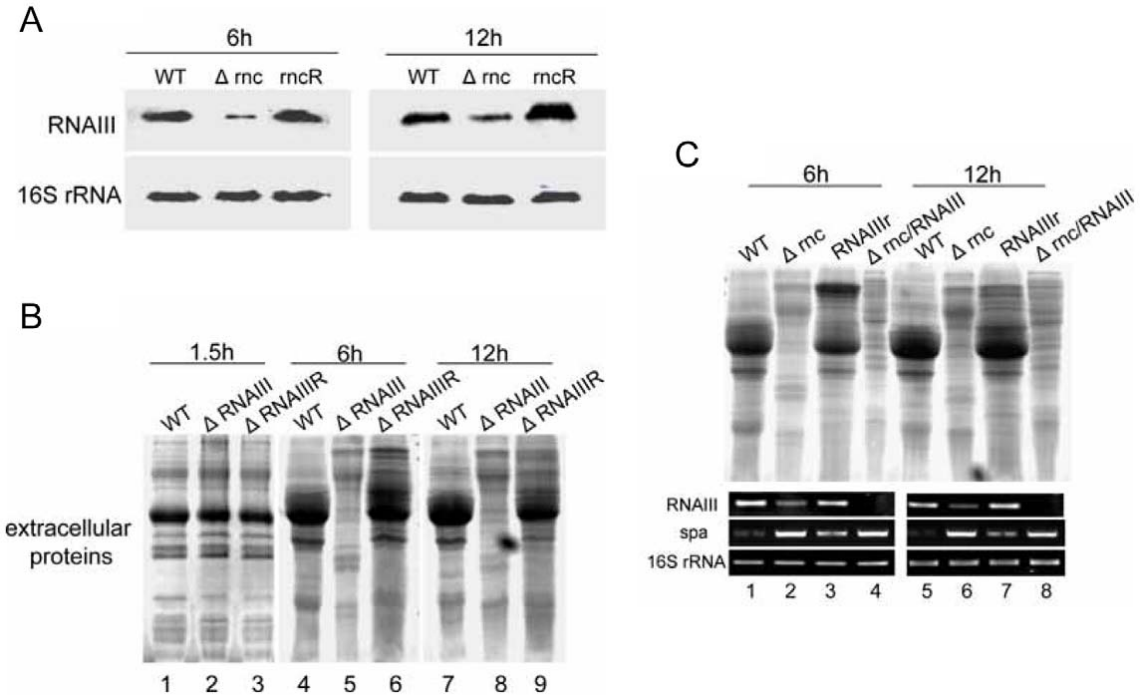
RNAIII regulates the levels of extracellular proteins at 6 h and 12 h. **A: The expression level of RNAIII was analyzed by Northern blot.** The level of RNAIII in different strains at 6 h and 12 h was detected by Northern blot. 16s rRNA was used as the internal control. WT: wild type, *S. aureus* 8325-4; *Δrnc*: an RNase III inactivation mutant from 8325-4; rncR: the restoration of RNase III activity in *Δrnc*. **B: Detection of the extracellular proteins of WT and** ***ΔRNAIII*** **at the different time points.** The extracellular proteins from the equal number of cells were extracted at the indicated time points. The results of SDS-PAGE showed that the extracellular proteins of *ΔRNAIII* were decreased in comparing with WT at 6 h and 12 h. 1,4,7: wild type; 2,5,8: *ΔRNAIII* (RNAIII deletion mutant); 3,6,9: ΔRNAIIIR (the restoration of RNAIII in *ΔRNAIII*). **C: Detection of the extracellular proteins from different strains.** The pOS1-RNAIII plasmid was constructed to recover the level of RNAIII in *Δrnc*. At the same time, the double mutant *Δrnc/RNAIII* was constructed. Then the extracellular proteins were extracted. The results showed that the extracellular proteins were increased at 6 h and 12 h after the level of RNAIII was recovered in *Δrnc*. The level of RNAIII was measured by RT-PCR. 16s rRNA was used as the internal control. 1,5: WT, wild type; 2,6: *Δrnc*; 3,7: RNAIIIr(the *Δrnc* strain transferred with the plasmid pOS1-RNAIII); 4,8, *Δrnc/RNAIII*. The experiment has been repeated for three times.

As RNAIII is a positive regulator of extracellular virulence [18] and RNase III can mediate the interaction between RNAIII and its target mRNAs [7], [8], we checked the level of RNAIII in *Δrnc* by Northern blot. Compared with its parent strain, the expression of RNAIII in *Δrnc* decreased at 6 h and 12 h (figure 3A). In order to avoid the unintended mutation in agr system during we constructed the *Δrnc*, we analyzed the sequence of agrA and agrC of *Δrnc*. No mutated nucleotide was observed in the genome of *Δrnc* strain (data not shown).

In the further study, we wondered whether the decrease of the extracellular proteins was due to the reduction of the RNAIII in *Δrnc*. The profile of the extracellular proteins of the *ΔRNAIII* (RNAIII deletion mutant) and its parent strain was tested at different time points. It was found that the extracellular proteins of *ΔRNAIII* decreased when compared with its parent strain at 6 h and 12 h (figure 3B). In the further investigation, the plasmid of pOS1-RNAIII was constructed and transferred into *Δrnc* to generate the strain of *RNAIIIr*, in which the RNAIII level was recovered. It was found that the extracellular proteins increased after the RNAIII level was recovered in *Δrnc* at 6 h and 12 h (figure 3C). The RNase III inactivated mutant was also constructed from *ΔRNAIII,* named as *Δrnc/RNAIII*. The profile of the extracellular proteins from *Δrnc/RNAIII* was the same as that from *ΔRNAIII* (figure 3C). Meanwhile, the levels of RNAIII and *spa* mRNA in the different strains were detected by RT-PCR (figure 3C). This results suggested that the lower level of RNAIII in *Δrnc* was responsible for the reduction of extracellular protein at 6 h and 12 h.

### The secretion of the proteins in *Δrnc* was inhibited at 1.5 h

**Figure 4:**
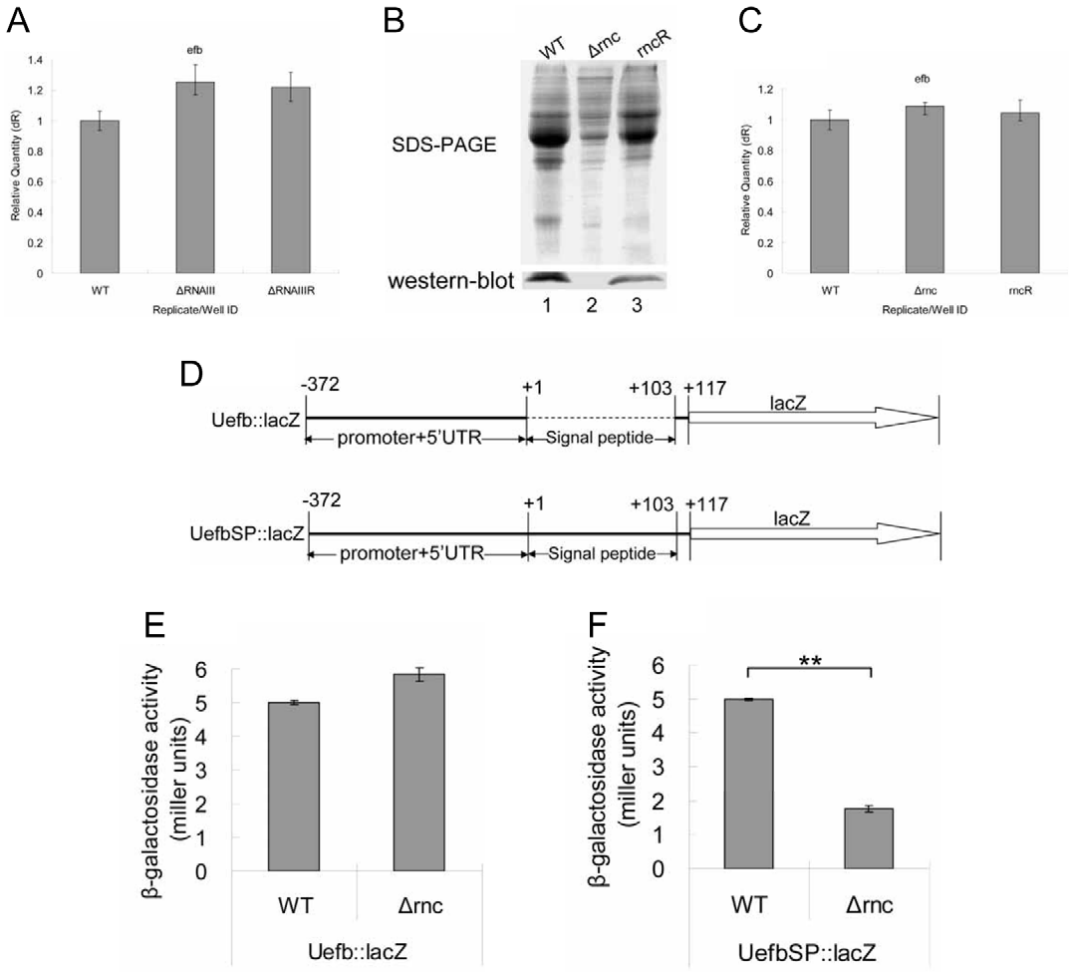
The secretion of the proteins in *Δrnc* was inhibited at 1.5 h. **A: qRT-PCR quantification of the level of** ***efb*** **mRNA.** The level of *efb* mRNA in the different strains was detected at 6 h. The results showed that the expression of *efb* was not regulated by RNAIII. WT: wild type; *ΔRNAIII*: RNAIII deletion mutant; ΔRNAIIIR: the restoration of RNAIII in *ΔRNAIII*. **B: Detection of the expression of Efb in the extracellular proteins from the different** ***S. aureus*** **strains by Western blot at 1.5 h.** The extracellular proteins from same number of cells were extracted from different *S. aureus* strains. The expression of Efb was tested with the specific antibodies of Efb (prepared by ourselves) by Western blot. The result showed that Efb couldn't be detected in the supernatant of *Δrnc*. 1: WT, wild type, *S. aureus* 8325-4; 2: *Δrnc,* an RNase III inactivation mutant from 8325-4; 3: rncR, the restoration of RNase III activity in *Δrnc*. **C: qRT-PCR quantification of the mRNA level of** ***efb*** **at 1.5 h**. The quantity of *efb* mRNA from different strains was measured by qRT-PCR at 1.5 h. The result showed that the level of *efb* mRNA wasn't changed in *Δrnc*. WT: wild type, *S. aureus* 8325-4; *Δrnc*: an RNase III inactivation mutant from 8325-4; rncR: the restoration of RNase III activity in *Δrnc*. **D: The schematic diagram of construction of the reporter vectors.** Uefb::lacZ: the promoter and 5′UTR of *efb* were fused with Lacz; UefbSP: the promoter, 5′UTR and the signal peptides of *efb* were fused with LacZ. **E: Detection of the β-galactosidase activity of different strains.** The Uefb::lacZ reporter vector was separately transferred into *Δrnc* and its parent strains. Then the β-galactosidase activity of different strains was measured at 1.5 h and expressed by miller units. There was no significant difference observed. The results represented a mean of three independent experiments. WT: wild type, *S. aureus* 8325-4; *Δrnc*: an RNase III inactivation mutant from 8325-4. **F:** **Detection of the β-galactosidase activity of the cultured medium from different strains**.The UefbSP::lacZ reporter vector was separately transferred into *Δrnc* and its parent strains. Then the β-galactosidase activities of the cultured medium were measured and expressed by miller units. Comparing with it parent stain, the β-galactosidase activitiy of the cultured medium from the *Δrnc* was decreased at 1.5 h. The results represented a mean of three independent experiments. WT: wild type, *S. aureus* 8325-4; *Δrnc*: an RNase III inactivation mutant from 8325-4. (**: p<0.01).

In above result there was no significant difference observed in the extracellular proteins production between *ΔRNAIII* and its parent strain at 1.5 h (figure 3B). The reason should be that RNAIII was a cell density-dependent regulator [19] and the level of RNAIII was too low to regulate its targets at the lag phase. However, the extracellular proteins in *Δrnc* decreased at 1.5 h (figure 2). It suggested that the reduction of extracellular proteins in *Δrnc* was not completely due to the RNAIII level decreasing. And the reduction of extracellular proteins in *Δrnc* at 1.5 h should have no relation with RNAIII. To discover other factors involved in this process, we chose Efb (extracellular fibrinogen binding protein) as the indicator of extracellular proteins [20], [21], [22] because the expression of Efb was not influenced by RNAIII (figure 4A) and the level of Efb in *Δrnc* supernatant decreased at 1.5 h (figure 4B).Then we analyzed the mRNA level, translation and secretion of Efb to discover the mechanism of reducing the extracellular proteins in *Δrnc* at 1.5 h. Firstly, the mRNA level of the *efb* gene was determined by qRT-PCR. It showed that the mRNA level of *efb* in *Δrnc* did not alter at 1.5 h (figure 4C). Secondly, we constructed the lacZ fusion vectors to analyze the translation of Efb in *Δrnc* and its parent strains (figure 4D). The upstream region of efb containing its promoter and 5′UTR was fused with lacZ (named as Uefb::lacZ). The constructed vector was transformed to *Δrnc* and its parent strain respectively. The results of β-galactosidase activity detection showed that *Δrnc* did not exhibit significant difference comparing with its parent strain (figure 4E). This suggested that the inactivation of RNase III did not influence the transcription and translation of Efb. And thirdly, we checked if the secretion of Efb was affected in *Δrnc*. The upstream of *efb* containing its promoter, 5′UTR and the signal peptide fused with lacZ was termed as UefbSP::lacZ (figure 4D). The β-galactosidase activity of cultured medium in the two different strains was detected. Our results showed that the β-galactosidase activity of the cultured medium from *Δrnc* was significantly lower than that from its parent strain (figure 4F). It suggested that the reduction of extracellular proteins of *Δrnc* at 1.5 h was because the secretion of extracellular proteins was suppressed.

### The decrease of *secY2* resulted in the inhibition of extracellular protein secretion in *Δrnc* at 1.5 h

**Figure 5:**
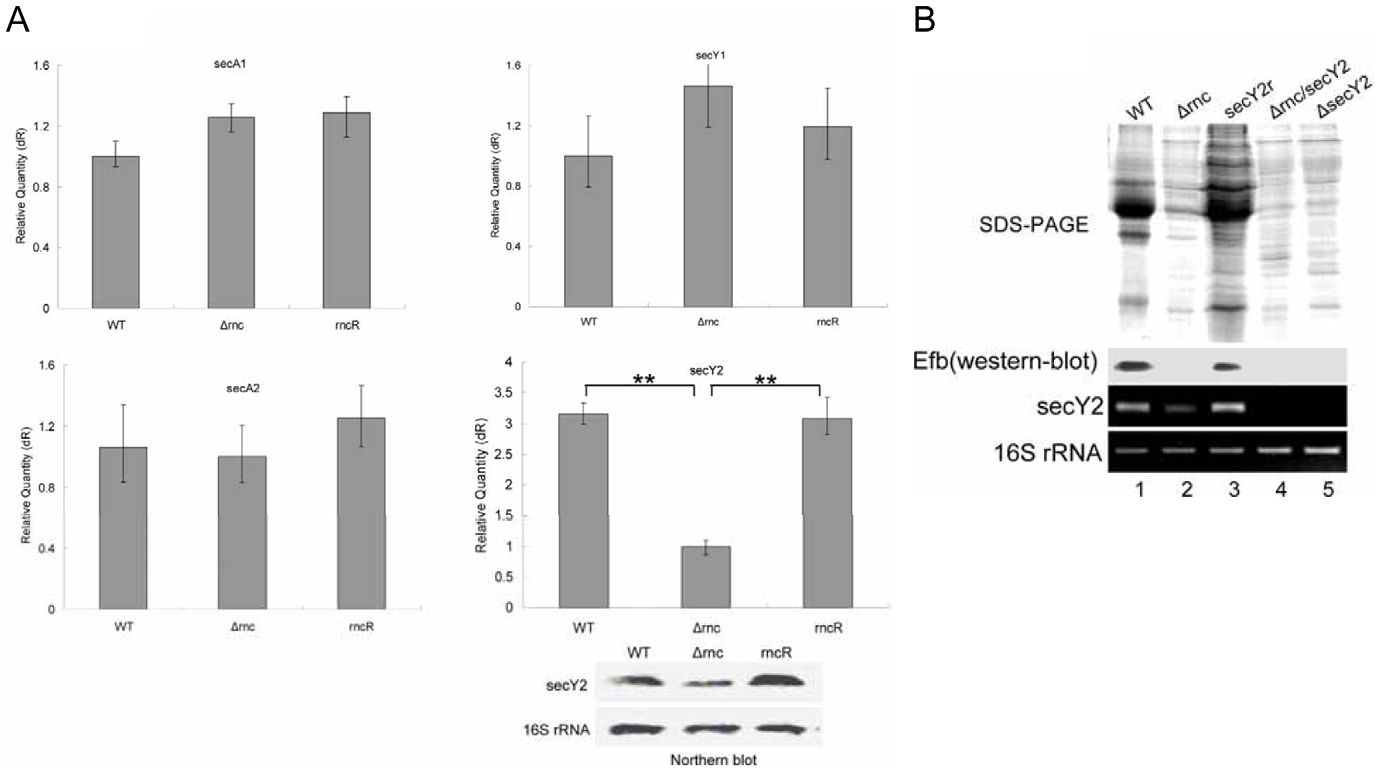
The decrease of *secY2* resulted in the inhibition of extracellular protein secretion in *Δrnc* at 1.5 h. **A: Detection of the mRNA level of** ***secA1, secY1*** **,** ***secA2*** **and** ***secY2.*** The mRNA levels of *secA1, secY1*,*secA2* and *secY2* were detected at 1.5 h by qRT-PCR. The results showed that the level of *secY2* was decreased in *Δrnc*. (**: P<0.01). Then the decrease of *secY2* mRNA was confirmed by Northern blot. 16s rRNA was used as the internal control. WT: wild type, *S. aureus* 8325-4; *Δrnc*: an RNase III inactivation mutant from 8325-4; rncR: the restoration of RNase III activity in *Δrnc*. **B: Detection of the profile of extracellular proteins and the expression of Efb in the different strains.** The pOS1-secY2 plasmid was constructed and transferred to *Δrnc* to recover the level of SecY2. At the same time, the double mutant *Δrnc/secY2* was constructed. And then the extracellular proteins were extracted. The results showed that the production of the extracellular proteins was significantly increased at 1.5 h after the recovery of the level of *secY2*. The mRNA level of *secY2* was measured by RT-PCR. 16s rRNA was used as the internal control. At the same time, the expression of Efb was determined by Western blot. The result showed that Efb was restored after the level of *secY2* was recovered in *Δrnc*. 1: wild type; 2: *Δrnc*; 3: secY2r(the *Δrnc* strain transferred with the plasmid pOS1-secY2); 4, *Δrnc/secY2;* 5. *ΔsecY2*.

The general secretory (sec) pathway is the most commonly used one for bacterial protein transport [12]. In addition, *S. aureus* contains an accessory Sec2 pathway involving the SecA2 and SecY2 proteins [13], [14]. However, there were few reports on the sec pathway of *S. aureus* [12]. We analyzed the genome of *S. aureus* and detected the mRNA level of the genes which were involved in the general and accessory sec pathway (*secA1*, *secY1*, *secA2* and *secY2*) by qRT-PCR. It was found that only the expression of *secY2* decreased significantly in *Δrnc* at 1.5 h (figure 5A). Then the decline of *secY2* mRNA level was confirmed by Northern blot (figure 5A). In additon, the production of extracellular proteins of *ΔsecY2* (SecY2 inactivation mutant) was decreased at 1.5 h compared with its parent strain (figure 5B). In the further study, we constructed the plasmid of pOS1-secY2 using the promoter of ssrA, which is a tmRNA in *S. aureus*. It was found that the level of ssrA in *Δrnc* was not altered (data not shown). And then the plasmid was transferred to *Δrnc* to recover the expression level of *secY2.* The result showed the extracellular proteins increased after the expression level of *secY2* recovered in *Δrnc* (figure 5B). At the same time, the level of Efb in the supernatant was correspondingly restored (figure 5B). In the further investigation, the RNase III inactivated strain was constructed from the *ΔsecY2* strain, named as *Δrnc/secY2*, it was found that the profile of extracellular proteins of the *Δrnc/secY2* was the same as that of *ΔsecY2* (figure 5B).

### The mRNA stability of *secY2* and *RNAIII* was decreased in *Δrnc*

**Figure 6:**
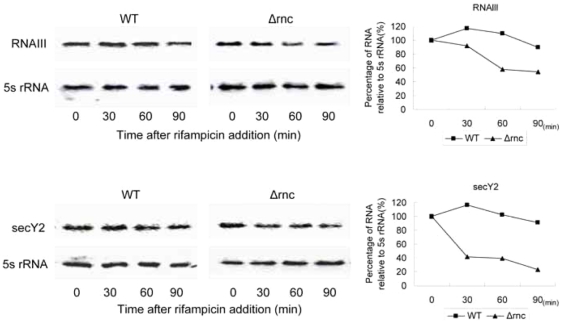
Stability of RNAs. Half-lives of *secY2* mRNA and RNAIII were determined in the presence of rifampicin (500 µg ml^-1^) in the WT and *Δrnc* strains. Percentage of RNA was calculated normalizing with 5s rRNA.

In order to investigate how RNase III influences the expression level of *secY2* and *RNAIII*, we tested the RNA stability of *secY2* mRNA and RNAIII. The transcriptions of *secY2* and *RNAIII* were inhibited by addition of rifampicin when the cells had been cultured at 37°C for 1.5 h or 6 h individually. Then the RNA stability was tested by Northern blot. It was found that RNA stability of *secY2* mRNA and RNAIII was decreased in *Δrnc* compared with its parent strain (figure 6).

### The *Δrnc* was less pathogenic compared with its parent strain

**Figure 7:**
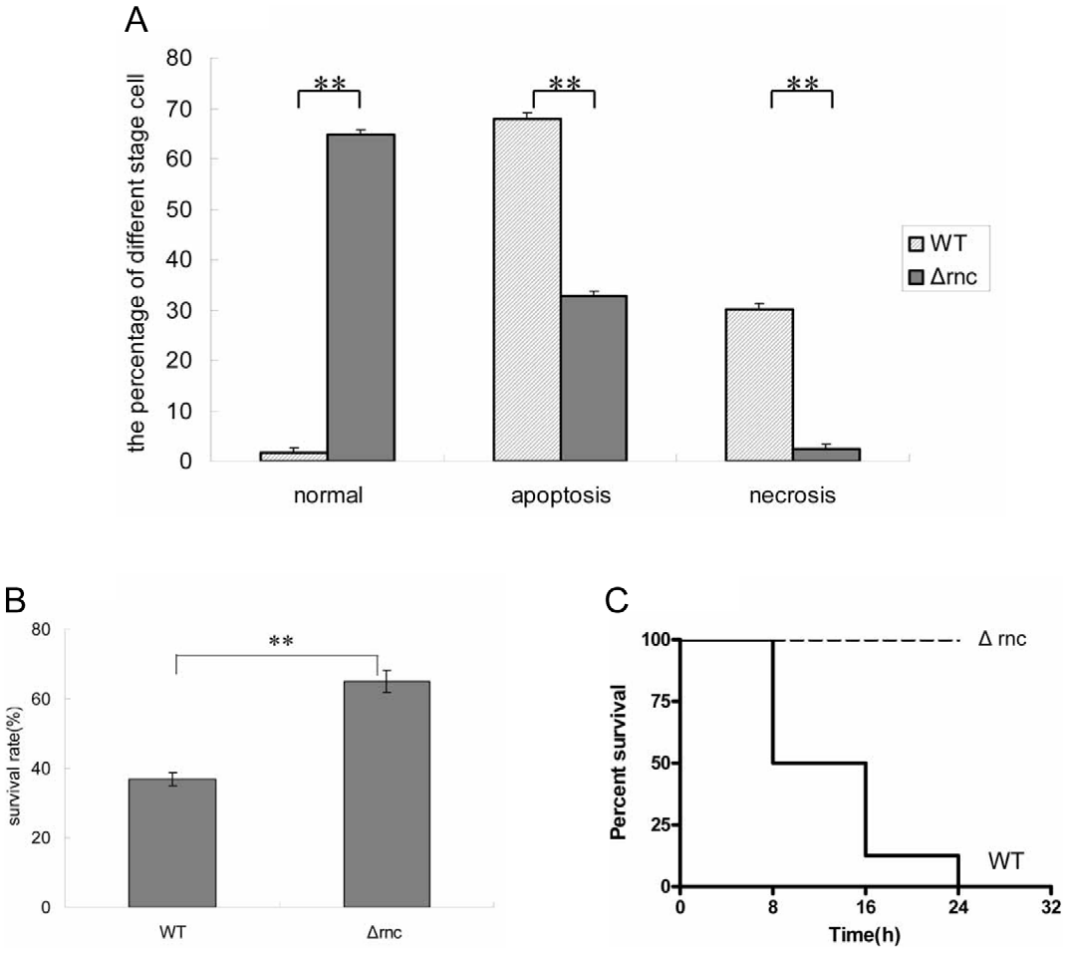
The *Δrnc* was less pathogenic compared with its parent strain. **A: Analysis of apoptosis and necrosis of MDBK cell after treatment with the supernatant from different strains.** Flow cytometric analysis was prepared to observe the apoptosis and necrosis of MDBK cell after treated with the supernatant of *S. aureus*. Comparing with its parent stain, the percentage of apoptosis and necrosis induced by the supernatant of *Δrnc* was significantly decreased. Data were from a representative experiment repeated for three times. (**: p<0.01). **B: Analysis of the production of the heat-stable toxins from different stains.** The heat-stable toxins were obtained as the described method and incubated with MDBK cell. The survival cell number was determined by MTT method. Comparing with its parent strain, the heat-stable toxins of *Δrnc* supernatant was decreased. Data were from a representative experiment repeated for three times. (**: p<0.01). **C: The detection of the pathogenicity of the different strains with the acute peritonitis animal model.** Groups of 10 Balb/c mice were injected intra-abdominally with 500 µl of *Δrnc* and its parent strain (1×10^8^ CFU). The number of the survival mice was recorded at different time points. The survival rate was calculated. The result showed that the pathogenicity of *Δrnc* was decreased.

In the further investigation, we compared the cytotoxicity induced by the supernatant between *Δrnc* and its parent strain. The supernatant of the cultured cells at 6 h was collected and incubated with MDBK cells. And then the cytotoxicity of the supernatant was tested with flow cytometric analysis. It was found that the percentage of apoptosis and necrosis induced by the *Δrnc* supernatant was significantly lower compared with its parent strain (figure 7A). At the same time, we also detected the cytotoxicity induced by the heat-stable toxins in the supernatant using MTT assay. In line with expectations, the heat-stable toxins of *Δrnc* were decreased (figure 7B). Then the pathogenicity of *Δrnc* was assessed in a murine peritonitis model. The same numbers of cells of *Δrnc* and its parent strain were delivered intraperitoneally to mice. As shown in figure 7C, the survival rate of the mice in the *Δrnc* group was significantly higher than that of its parent strain group at the different time points (8 h, 16 h, and 24 h), which was in accordance with the cell toxicity results. It suggested that the RNase III played an important role in the pathogenicity of *S. aureus.*

## Discussion

As we know, the extracellular proteins play an important role in the infection caused by *S. aureus* [11]. In our study, we find that the extracellular proteins in the supernatant of *Δrnc* decreased significantly at three different growth phases compared with its parent strain.

Most of the extracellular virulence factors in *S. aureus* is positively regulated by a regulatory RNA molecule — RNAIII [23], [24], which is thought as an antisense RNA regulating many genes expression (except δ-hemolysin encoding). It is demonstrated that the duplex of mRNA and RNAIII can be degraded by RNase III *in vitro* [7], [8].

We find that the expression of *spa*, one target RNA of RNAIII which can be degraded by RNase III, increased in *Δrnc*. It indicates that the degradation mediated by RNAIII is altered. In the further investigation, it shows that the level of RNAIII decreased in *Δrnc*. These results suggest that the decline of RNAIII may be the reason for the reduction of extracellular proteins of *Δrnc*. In our experiments, it is revealed that the extracellular protein of *ΔRNAIII* decreases comparing with its parent strain at 6 h and 12 h. After the level of RNAIII is recovered in *Δrnc*, the extracellular proteins increased correspondingly. In the meantime, the reduction of extracellular proteins of *Δrnc* at 1.5 h should not be due to the decrease of RNAIII because the level of RNAIII is cell density dependent and too low at 1.5 h. The profiles of extracellular proteins among *Δrnc*, *Δ*RNAIII and *Δrnc/*RNAIII are similar, but the density of several bands is different. These results suggest that there should be some other proteins regulated by RNase III through the RNAIII- independent pathway. Two previous reports showed that the level of RNAIII was not altered in the *rnc* mutant strains from RN6390, which is not consistent with our results [7], [8]. The occurrence of the different results might be due to the different strains used in the experiments. Our previous work also has shown that there are some difference between *S. aureus* 8325-4 and RN6390, although both of them are originated from *S. aureus* 8325 [25], [26], [27], [28].

In the investigation, it is also found that the extracellular protein Efb decreases at 1.5 h in the supernatant of *Δrnc*. Efb is indentified as the 15.6-kilodalton extracellular fibrinogen-binding protein [20], [21], [22]. Hence, we chose Efb as an indicator for the further study. Our results show that the transcription and translation of Efb are not altered in *Δrnc*. However, the results of β-galactosidase activity assay show that the secretion of β-galactosidase decreases in *Δrnc* supernatant when lacZ gene is fused with the Efb signal peptide. These results suggest that the transport of Efb is inhibited in *Δrnc*.

The secretory (sec) pathway, which includes sec1 and sec2 in *S. aureus*, is thought to be responsible for secretion of the extracellular proteins of *S. aureus*, but there are few published data on the sec pathway of *S. aureus* [12]. So we checked the expression of the components of sec pathway by qRT-PCR. It is found that the level of *secY2* significantly decreases in *Δrnc*. The further investigation shows that the recovery of secY2 in *Δrnc* can increase the production of extracellular proteins at 1.5 h. In several pathogenic gram-positive bacteria, the accessory sec pathway (SecA2 and SecY2) is required for the transport of certain proteins related to virulence[12], [15], [16], [17]. In addtion, it has been reported that SecY2 can interact with the Sec1 channel in *S. aureus* [13]. Our results show that Sec2 is the major pathway which is responsible for the extracellular proteins transport of *S. aureus* at 1.5 h. It is interesting that we do not observe significant difference of the extracellular proteins profile between *ΔsecY2* and wild type at 6 h and 12 h (data not shown). Our data indicates that SecY2 might play an important role in protein secretion at lag phase but not at exponential and stationary phases.

We also found that the stability of *secY2* mRNA and RNAIII was decreased in *Δrnc*. It suggests that RNase III is involved in the RNA degradation of both genes. RNase III is a kind of ribonuclease, so the decline of the RNA stability in *Δrnc* should be indirectly regulated by RNase III. We are trying to investigate the mechanism of this regulation.

In conclusion, our study reveals a novel biological function of RNase III in *S. aureus*, which can regulate the production of extracellular proteins via two molecules respectively at the different growth phases. At the lag phase, RNase III can positively regulate the level of *secY2* to increase the secretion of the extracellular proteins. After *S. aureus* cells grow to a certain density, RNase III can regulate the expression of extracellular proteins by affecting the level of RNAIII. Since the extracellular proteins are essential for the infection caused by *S. aureus*, RNase III might be a potential target of anti-Staphylococcus aureus infection.

## Materials and Methods

### Ethics Statement

This study was carried out in strict accordance with the recommendations in the national guidelines for the use of animals in scientific research “Regulations for the Administration of Affairs Concerning Experimental Animals”. The protocol was also approved by the Animal Care and Use Committee of Beijing Institute of Basic Medical Sciences (Permit Number BMS-091008), and all efforts were made to minimize suffering.

### Bacterial strains and growth conditions

The strains used in this study are listed in Table 2. Strains were grown in 5 ml of brain heart infusion (BHI) or Luria-Bertani(LB)medium (BD) at 37°C for 12 h with shaking at 200 rpm in a 25-ml test tube. Cells from 1 ml of preculture were transferred to 100 ml of BHI or LB medium in a 500-ml flask and incubated at 37°C on a rotary shaker at 200 rpm. *S. aureus* strains were routinely grown in BHI and *E. coli* strains were grown in LB medium either without antibiotics, or with 20 µg ml^−1^ erythromycin, 100 µg ml^−1^ ampicillin and 100 µg ml^−1^ kanamycin respectively.

**Table 2:** Bacterial strains and plasmids.

Strain or plasmid	Comments	Source or reference
**Strain**		
***S. aureus***		
8325-4	Wild-type, rsbU^-^	[25]
RN4220	Restriction-negative strain, 8325 derivative	[35]
*Δrnc*	8325-4 with a rnaseIII::kan mutation	This study
rncR	the restoration of RNase III activity in *Δrnc*	This study
*ΔRNAIII*	8325-4 with a rnaIII::kan mutation	This study
*Δ*RNAIIIR	the restoration of RNAIII activity in *ΔRNAIII*	This study
*ΔsecY2*	8325-4 with a SecY2 inactive mutation	This study
*Δrnc/secY2*	a secY2 and RNase III double inactive mutation	This study
*Δrnc/RNAIII*	a RNAIII and RNase III double inactive mutation	This study
***E. coli***		
DH5α	A host strain for cloning	Transgene
**Plasmids**		
pAUL-A	Temperature-sensitive *S. aureus* suicide vector; Em^r^	[36]
pMD19T	*E. coli* cloning vector, amp^r^	TaKaRa
pOS1	*E. coli*-*S. aureus* shuttle vector, Cm^r^	[37]
pOS1-RNAIII	pOS1 derivative for expression of *RNAIII*	This study
pOS1-secY2	pOS1 derivative for expression of *secY2*	This study
pOS1-lacZ	pOS1 contains a copy of lacZ encoding β-galactosidase without promoter and 5′UTR	This study
pOS1-Uefb-lacZ	UTR of *efb*-lacZ fusion(Uefb::lacZ) shuttle vector, a derivative of pOS1	This study
pOS1-UefbSP-lacZ	UTR and signal peptide of *efb*-lacZ fusion(UefbSP::lacZ) shuttle vector, a derivative of pOS1	This study

### Construction of insertion mutant of RNase III (*Δrnc*)

The mutant was constructed using the procedures described previously [29] with some modifications. In order to create an insertion mutant of RNase III in *S. aureus* 8325-4, two regions of DNA flanking the *rnase III* gene were amplified by PCR using the primers (Up-*rnc* F-EcoRI/Up-*rnc* R-KpnI, Down- *rnc* F-KpnI/Down- *rnc* R-SalI) with restriction sites as listed in the Table 1. The upstream fragment (529 bp) was digested with *Eco*RI and *Kpn*I, and the downstream fragment (232 bp) was digested with *Kpn*I and *Sal*I. The two fragments were cloned together into pMD19T digested with *Eco*RI and *Sal*I. The resulting construct was digested with *Kpn*I, and then a 1.6-kb kanamycin cassette which was amplified from the plasmid of pTZ-TRAP::kan provided by Dr. Balaban N was inserted. The resulting plasmid was digested with *Eco*RI and *Sal*I, and a fragment harboring kanamycin resistance between the upstream and downstream fragments was ligated into pAUL-A digested with *Eco*RI and *Sal*I to create plasmid pAUL-A-rnc. pAUL-A has a temperature-sensitive origin of replication that is active in *S. aureus* at 30°C but not at 42°C. The recombinant plasmid, initially isolated from *E. coli*, was introduced into *S. aureus* RN4220 by electroporation and colonies resistant to kanamycin and erythromycin were selected after growth at 30°C. The resistant clones were subjected to a temperature shift to 42°C to select for plasmid integration into the chromosome. Bacteria resistant to kanamycin but sensitive to erythromycin were selected. The mutation was confirmed by PCR, and followed by transduction into strains 8325-4 with phageΦ11 to create strains *Δrnc* in which RNase III was inactive (Table 2).

### Restoring RNase III Activity

Primers rs-rncF and rs-rncR (listed in Table 1) were designed to PCR-amplify an 1045 bp fragment encompassing the gene encoding RNase III, its promoter region and termination site and *Sal*I/*Eco*RI sites, using *S. aureus* 8325-4 chromosomal DNA as template. The PCR product (“whole *rnase III*”) was digested and cloned into the *Sal*I/*Eco*RI digested pOS1, which could replicate in *E. coli* and in *S. aureus* at 37°C. The resulting plasmid (pOS1-rs- rnase III) was used to transform into *E. coli* DH5α by electroporation. Cells were selected on LB plates containing 100 µg mL^−1^ ampicillin. The combinant plasmid was isolated from positive clones and used to transform *S. aureus* RN4220 cells, and transformants were selected on tryptic soy agar plates containing 10 µg ml^−1^ chloromycetin at 37°C. A positive clone (RN4220 containing pOS1-rs-rnase III) was selected by colony PCR using primers rs-rncF and rs-rncR. Then the plasmid was isolated and transformed to *Δrnc* by electroporation. The transformants were selected on tryptic soy agar plates containing 10 µg ml^−1^ chloromycetin at 37°C for 20 h. The restoring colonies (*rncR*) were confirmed by PCR and RT-PCR analysis.

### Growth curve assay

To measure growth curves, a colony was picked from a plate of *S. aureus* 8325-4 and *Δrnc* and then inoculated in 5 ml of BHI media at 37°C for 14 h on a rotary shaker at 200 rpm. Cell densities were measured at OD600_nm_ using BHI media as a blank. The culture was diluted to achieve an OD600_nm_ of 0.1 in 10 ml. Then the bacteria were incubated at 37°C at 200 rpm, and cell densities were determined at OD600_nm_ every hour.

### Proteins analysis by sodium dodecyl sulfate-polyacrylamide gel electrophoresis (SDS-PAGE)

The cell extract was prepared as described [30] with some modifications as follows. Cells were grown with shaking at 37°C at 200 rpm. Equal numbers of cells were collected at indicated time and then resuspended in 100 µl Tris-EDTA buffer containing lysostaphin (100 µg ml^−1^, Sigma-Aldrich). After incubation for 30 min at 37°C, the mixture was sonicated on ice three times for 30 s each. Protein samples (15 µl) were mixed with SDS-PAGE loading buffer and then subjected to 15% SDS-PAGE. Gels were stained with Coomassie blue G-250 (Sigma-Aldrich).

Extracellular protein profiles were determined as described [31], [32] with some modifications as follows. Briefly, *S. aureus* cells were grown at 37°C and growth culture was centrifuged at 6,000 g for 10 min at 4°C. The supernatant was collected and filtered through a 0.22 µm filter to remove residual cells. Culture supernatant from equal numbers of cells was precipitated by adjusting filtered supernatants to 10% tricarboxylic acid (TCA) and incubated at 4°C for 4 h. After centrifugation (12,000 g, 10 min), precipitated proteins were washed twice in ice-cold 96% ethanol, air dried. The proteins were resolved in an appropriate volume of a solution containing 7 M urea, 2 M thiourea. The samples were then subjected to 15% SDS-PAGE and visualized by Coomassie blue G-250 staining.

### Flow cytometric analysis of apoptosis and necrosis of MDBK cells

The supernatant was collected according to the above method. For flow cytometric analysis, the MDBK cells were resuspended at a concentration of 1×10^6^ cells ml^−1^ and added to a 12-well plate (1 ml/well). At 40–50% confluency (24 h post seeding), the cultivated cells were treated with medium alone or with supernatant of *S. aureus* strains for 12 h. Prior to harvesting, the cells were washed twice with PBS (Phosphate Buffered Saline), trypsinized, and pelleted. Then cells were resuspended at a concentration of 1×10^6^ cells ml^−1^ in Binding Buffer (0.01 M HEPES/NaOH, pH 7.4, 14 mM NaCl, 0.25 mM CaCl_2_). Aliquot cells (500 µl) were added into FACS tubes and mixed with 25 ng ml^−1^ FITC-conjugated annexin V and 10 mg ml^−1^ propidium iodide(PI), incubated for 15 min at room temperature in the dark. Then the apoptosis and necrosis were analyzed immediately by flow cytometry. The final data was reported as the mean ± SD for each of the three independent experiments.

### MTT assay of cellular toxicity

For MTT assay, the supernatant was boiled for 10 min, and then centrifuged to remove the precipitate. The MDBK cells were cultured in RPMI-1640 medium (Gibco) with 100 U ml^−1^ penicillin and 100 µg ml^−1^ streptomycin at 37°C with 5% CO_2_. Then they were detached using 0.25% trypsin/EDTA and counted by means of hemocytometer. The cells were resuspended and a total of 1×10^4^ in 0.1 ml culture medium was seeded into each well of a 96-well plate and cultured for 24 h. At 40–50% confluency (24 h post seeding), the cultivated cells were treated with medium alone or with the boiled supernatant of *S. aureus* strains. Then, MTT assay was performed 24 h after treatment. 10 µl of MTT (1 mg ml^−1^, Sigma-Aldrich) was added into each well and the incubation was continued for 4 h at 37°C with 5% CO_2_. After 4 h, 100 µl SDS buffer (10%SDS, 0.1 M HCl) was added to the wells. The absorbance of the wells was determined using a plate reader at a test wavelength of 595 nm after 8 h. The cell viability percentage was calculated as: Viability percentage (%)  =  (Absorption value of supernatant of treatment group)/(Absorption value of supernatant of control group) ×100%.

### Western blot

The protein samples were subjected to 15% SDS-PAGE and the proteins were blotted onto Hybond-ECL nitrocellulose membrane (Amersham Biosciences). The membrane was blocked in 5% non-fat dry milk at 37°C for 2 h, probed with1∶500 diluted polyclonal rabbit anti-Efb antibodies (prepared by ourselves) for 1 h at room temperature, and washed twice in PBS with 0.5% Tween 20 (PBST). Then the membrane was incubated in a 1∶5,000 solution of HRP-conjugated goat anti-rabbit secondary antibody at room temperature for 1 h. After further washing with PBST, the membrane was assayed by the enhanced chemiluminescence (ECL) western blotting detection system.

### Quantitative reverse transcription PCR (qRT-PCR)

Total bacterial RNA was extracted from *S. aureus* using Trizol (Invitrogen) as previously described [30]. DNase digestion of 80 µl of total RNA was performed with 10 U of RNase-free DNase I (Promega) and 10 µl of the 10× reaction buffers in a total reaction volume of 100 µl for 30 min at 37°C. For cDNA synthesis, 1 µg of total RNA was mixed with 500 ng of random hexamer (Promega). Samples were incubated at 65°C for 10 min with 5 µl of 5× first-strand buffer, 2 µl of 5 mM dNTP, 20 U of RNasin (Takara), 1 µl of M-MLV reverse transcriptase (Promega) and distilled water to a total volume of 25 µl. The qRT-PCR reaction mixture contained 12.5 µl of 2×SYBR green PCR mix (GenePharma), 0.3 µM of gene-specific forward and reverse primers, and 1 µl of template, made up to a final volume of 25 µl with distilled water. The primers are shown in Table 1. Cycling parameters were set as follows: initial activation step at 95°C for 10 min, denaturation at 94°C for 30 s, annealing at 58°C for 30 s, and extension at 72°C for 40 s. Melting curve analysis was performed from 58°C to 95°C with stepwise fluorescence acquisition at every 1°C s^−1^. Melting curves observed for each gene were confirmed to correspond to the correct amplicon size by agarose gel electrophoresis of the PCR products. The levels of gene expression were calculated by relative quantification using 16s rRNA as the endogenous reference gene. All samples were amplified in triplicate and the data analysis was carried out using the MxPro qRT-PCR system software (Stratagene).

### Construction of lacZ reporter vector

The fragments (promoter-5′UTR and promoter-5′UTR-signal peptide of *efb*) was amplified by PCR from *S. aureus* 8325-4 chromosomal DNA with primers Uefb-lacZF/Uefb-lacZR and Uefb-lacZF/UefbSP-lacZR (Table 1). The PCR products were digested with *Eco*RI and *Bam*HI, and ligated into *EcoR*I and *Bam*HI-digested pOS1-lacZ plasmid DNA, which contains a copy of lacZ without promoter and 5′UTR, resulting in the in-frame fusion of lacZ to the amplified fragments. The recombinant plasmids were transformed into DH5α, then electrotransfected to *S. aureus* RN4220. The plasmid was isolated from RN4220, then electrotransfected into *S. aureus* 8325-4 and *Δrnc*.

### β-Galactosidase assay

The cells were prepared for the assay as described before with some modification [33]. Cells were grown as described above, and 1 ml culture was centrifuged. Briefly, the pellet was washed in PBS, and then the cells were adjusted to an OD600_nm_ of 1 in a volume of 500 µl. The cells were sedimented by centrifugation and the pellet was resuspended in 500 µl lysis buffer (0.01 M potassium phosphate buffer, pH 7.8, 0.015 M EDTA, 1%Triton X-100) containing lysostaphin at the final concentration of 20 µg ml^−1^, and incubated at 37°C for 30 min, with gentle shaking. The culture was centrifuged at 20,000 g for 30 min. The supernatant was subjected to galactosidase assays according to the method described by Miller [34].

### Northern Blot and RNA half-life determination

Total RNA was separated by electrophoresis on a 1.2% agarose gel containing 2.2 M formaldehyde and transfered to nylon membrane. Hybridizations with the specific α-^32^P-labeled DNA probes were carried out to detect the *secY2* mRNA or RNAIII. 16s or 5s rRNA was used as the internal control.

RNA half-lives were determined by treating cells with rifampicin (final concentration: 500 µg ml^−1^) and isolation of RNA at 0,30, 60, and 90 min after rifampicin addition. *SecY2* mRNA stability was determined in lag phase (cultured for 1.5 h; OD600 = 0.5) and RNAIII stability was was determined in stationary phase (cultured for 6 h; OD600 = 10).

### Acute murine peritoneal infection model

Groups (n = 10) of 6- to 8-week-old, male Balb/c mice were injected intra-abdominally with 500 µl of *Δrnc* or its parent strain (containing 1×10^8^ CFU (colony forming units)). The survival number of mice was recorded at the different time points (8 h, 16 h and 24 h) post challenge. Survival outcomes in *Δrnc* or its parent strain groups were compared. The experiment was performed twice.

### Statistical analysis

All quantitative data were analyzed using student t-tests. P<0.05 was considered statistically significant.
